# Corrosion Behaviour of High-Strength Al 7005 Alloy and Its Composites Reinforced with Industrial Waste-Based Fly Ash and Glass Fibre: Comparison of Stir Cast and Extrusion Conditions

**DOI:** 10.3390/ma14143929

**Published:** 2021-07-14

**Authors:** Praveen Kumar Swamy, Shantharaja Mylaraiah, Manjunath Patel Gowdru Chandrashekarappa, Avinash Lakshmikanthan, Danil Yurievich Pimenov, Khaled Giasin, Munishamaiah Krishna

**Affiliations:** 1 Department of Mechanical Engineering, University Visvesvaraya College of Engineering, Bengaluru 560056, India; shantharajam@gmail.com; 2 Department of Mechanical Engineering, PES Institute of Technology and Management, Shivamogga, Visvesvaraya Technological University, Belagavi 590018, India; 3 Department of Mechanical Engineering, Nitte Meenakshi Institute of Technology, Bengaluru, Visvesvaraya Technological University, Belagavi 590018, India; avinash.laks01@gmail.com; 4 Department of Automated Mechanical Engineering, South Ural State University, Lenin Prosp. 76, 454080 Chelyabinsk, Russia; danil_u@rambler.ru; 5 School of Mechanical and Design Engineering, University of Portsmouth, Portsmouth PO1 3DJ, UK; Khaled.giasin@port.ac.uk; 6 Department of Mechanical Engineering, RV College of Engineering, Bengaluru 560059, India; krishna_phd@yahoo.co.in

**Keywords:** extrusion, gravimetric, electrochemical impedance, corrosion rate, Al 7005 composites, S-glass fibre, fly ash

## Abstract

The stringent demand to develop lightweight materials with enhanced properties suitable for various engineering applications is the focus of this research work. Industrial wastes such as fly ash (FA) and S-glass-fibres (GF) were used as reinforcement materials for high-strength alloy, i.e., Al 7005. Stir casting routes were employed for fabricating the four samples, Al 7005, Al 7005 + 5% GF, Al 7005 + 6% FA and Al 7005 + 5% GF + 6% FA. The extrusion process with different extrusion ratios (ER: 5.32:1, and 2.66:1) was used to examine the properties of all four samples. Extruded samples with ER: 5.32: 1 resulted in equiaxed grains with refined structure compared to stir casting parts. The effect of the extrusion process and the addition of reinforcements (GF and FA) on the gravimetric, electrochemical, and electrochemical impedance corrosion behaviour of Al 7005 composites in 1M HCl (Hydrochloric acid) solution were investigated. The results of all three corrosion methods showed that Al 7005 + 6% FA exhibited higher corrosion resistance. Corrosion rate of Al 7005, Al 7005 + 5% GF, Al 7005 + 6% FA and Al 7005 + 5% GF + 6% FA is found equal to 3.25, 2.41, 0.34, and 0.76 mpy, respectively. The FA particles remain inert and act as a physical barrier with corrosive media during the corrosion test. GF undergoes fibre degradation or disrupts the continuity of the glass network as a result of fibre leaching, which increases the corrosion rate in the sample. The gravimetric study showed that the corrosion rates decreased with an increase in extrusion ratio, which might be due to corrosion passivation increases and improved properties. The scanning electron microscopy reveals that corrosion fits, flakes and micro-cracks were observed more in the as-cast composites than that of extrusion composites, promoting the corrosion rate.

## 1. Introduction

Composite materials often combine two or more different materials (metal/ceramic/nonmetal) to combine lightweight with superior performance in fabricated parts, suitable for engineering (automotive, aerospace, marine, biomedical) applications [1,2,3,4,5]. Metal matrix composites (MMCs) are at the forefront in fabricating engineered parts due to their excellent properties (physical, mechanical, and electrical) and offer service even at elevated temperatures [6,7,8]. For MMCs, selecting the cost-effective processing route (liquid state processing: casting processes, powder metallurgical: pressing, sintering, extrusion, forging, or joining) to fabricate parts with enhanced properties is of industrial relevance [9]. The properties of MMCs are more sensitive to morphology, type and size of reinforcements, and method of fabrication [10,11,12,13]. Extruded parts (Aluminium and Magnesium-based composites) resulted in high tensile strength and refined microstructure properties compared to casted components [14,15]. Significant attention is thus required for the development of novel lightweight composites through extrusion routes, suitable for engineering applications.

7XXX series Al alloy possesses intrinsic properties such as high strength-to-density ratio, high-specific stiffness, and toughness characteristics making it an ideal candidate material for structural parts in automotive and aerospace applications [16,17]. The addition of reinforcement (organic, inorganic, industrial and agriculture waste, carbides, nitrides, oxides, CNTs, compounds) to aluminium matrix resulted in multiphase materials (composite materials) which improve the specific properties and attract engineering applications [18,19,20,21]. Silicon carbide reinforcements with different sizes and volumes, added to Al-Cu-Mg alloy resulted in improved strength in composite parts [22]. Table 1 illustrates the addition of reinforcements to aluminium and its alloy resulted in improved properties (hardness, strength, and wear resistance) in extruded parts. The extrusion process ensures uniform dispersing of reinforced nanoparticles in the matrix and thereby results in enhanced properties with refined grain structure in aluminium metal matrix composites [23,24,25]. The application of hard reinforcement particles, i.e., carbides, resulted in extrusion die wear [26,27]. Reinforcing hard materials into the matrix resulted in many manufacturing problems (tool wear in machining, resistance to deformation in forming process) [28,29]. The extrusion parameters (such as temperature and extrusion ratio) affect the microstructure and mechanical strengths in extruded parts [24,30,31]. Table 1 illustrates that there are different extrusion ratios employed by distinguished researchers, which dictates there exists a significant scope to conduct an intense experimental study. An increase in extrusion ratio results in a reduction in cross-section [24], whereas an increase in extrusion temperature results in a proportional decrease in the force required to fabricate the parts [30]. The coalescent cracks tend to grow rapidly by connecting with each other which resulted in lower strength beyond the critical extrusion ratio [24]. Therefore, the study of extrusion parameters and alternate reinforcing materials that result in ease of fabrication of extruded parts with enhanced properties at reduced costs is of industrial relevance.

In search of alternate materials, the use of tonnes of agro-industry wastes (fly ash) and composite waste (carbon and glass fibres) could result in reduced negative environmental impact and cost of reinforcement materials [32,33]. In 2017, the study conducted by the ministry of India estimated that the fly ash generation is approximately 300 million tons [33]. Wind turbine blades fabricated through glass fibre-reinforced plastics have an expected life span of 20 years. The survey predicted that by 2030, approximately 100,000 tons/year will be accumulated with wind turbine blades [32,34]. In addition, printed circuit boards and other electronic products fabricated viz. glass fibre-reinforced composites cumulatively end up generating 27.4–45.55 wt.% of glass fibres [32,35,36]. In the United Kingdom, generated waste glass fibres are recycled and reused up to 6% and the rest of the materials are treated waste, therefore ending up in landfill [37].

Rapid progress in the development of polymer composites led to the development of various engineering applications. Environmental concerns also demand useful applications for effective recycling of glass fibre and fly ash as potential reinforcements materials for fabricating parts for the said applications. Reinforcing glass fibre to a metal matrix (aluminium and zinc–aluminium) resulted in enhanced strength and hardness in the fabricated composites [38,39]. Al 7075 reinforced with glass fibres improved the damping behaviour of composites [40]. Industry wastes such as E-glass and fly ash particles were reinforced to Al 6061 and Al 6063 alloys to fabricate composites via the stir casting route [41]. Note that fly ash with 6% wt. to aluminium alloys (Al 6113, Al 6061 and Al 6063) resulted in improved hardness, wear-resistance and microstructure properties [41,42]. The above literature review confirms that limited studies have been carried out with industrial wastes as potential reinforcement materials for fabricating composites, although reinforcement particles offer beneficial properties.

Al 7XXX series alloys are ultra-high-strength materials and therefore 70% of materials are used for structural applications in aircraft [16,50]. It was observed that 60% of structural parts are fabricated viz. extrusion, 28% by rolling, 7% by forging and 5% by casting routes [16]. However, next-generation materials need to enhance the hardenability, damage tolerance and corrosion resistance in Al 7XXX alloys [16,51]. Although aluminium material possesses good mechanical properties, it becomes corroded rapidly [52]. Due to the combined effect of the operating environment and bearing loads during their service, stress corrosion is always seen to have a fatal defect in structural materials (in particular Al 7XXX alloys) that causes aircraft accidents [53]. The extrusion process refines the grain structure due to increased plastic deformation as a result of fracture of reinforcement particles to finer sizes which stimulates dynamic recrystallization and nucleation [24,25,54]. The microstructural change influences on corrosion behaviour of the alloys. An increase in SiC reinforcements with reduced particle size improves the corrosion resistance in aluminium alloy, as a result of the change in microstructure [55]. The polarization resistance plays a vital role in metallic corrosion subjected to test samples exposed to a corrosive environment. Note that, corrosion kinetics in active metal is more predominant than passive metals under corrosive environments. This is because pitting action breaks the protective passive films resulting in the initiation of corrosion on metal surfaces. The refined grain or microstructure results in a reduced corrosion rate in an extruded magnesium-based MMCs [56,57]. Although a lot of research efforts are being made on improving Al 7XXX alloy properties, less attention is paid to enhancing corrosion resistance properties.

Al 7005 alloy possesses intrinsic properties such as high strength, plasticity, weldability, with lightweight characteristics ensures widely applied in aircraft, marine ships, and rail transportation parts [58]. High-speed trains, aircraft and marine ships require load-bearing properties to minimize the incidence of parts failure against stress corrosion cracking and are subjected to a corrosive environment [59,60,61]. In general, S-containing species are generated viz. chemical reactions take place between sulphur dioxide and water in marine atmosphere [59,62]. This could destroy the passive film of aluminium alloy and acidification of electrolyte film (if any) on the material surfaces [63,64]. At present, hybrid composite materials are often being used for various engineering applications, due to enhanced properties with the use of dual reinforcements. There is increased hardness with short glass fibres and compression and ductility with reinforcing fly ash reinforcements. In general, hybrid composites consist of n (n > 2) jointly working phases, because they impart high strength resulting from different phases. Enhancing corrosion resistance property for high strength aluminium alloy is indeed essential and could widen the applications.

The novelty of the present work is defined to limit the corrosion rate of Al 7005 subjected to a corrosive environment, with the aim of the following: (a) use of industrial wastes (fly ash and S-glass fibres) as a cost-effective reinforcement material for fabricating Al 7005 hybrid composites. (b) Study of microstructure of four tests samples (as-cast Al 7005, extruded: Al7005 + 5% GF, Al7005 + 6% FA, Al7005 + 5% GF + 6% FA). (c) Study of different extrusion ratios on the corrosion rate of all four test samples 1M HCl solutions at different exposure durations using gravimetric corrosion studies. (d) Study of different extrusion ratios on the corrosion rate of test samples in 1M HCl solutions, using electrochemical corrosion tests (polarization curves and electrochemical impedance spectra). (e) Comparison of the morphologies of microstructures with and without samples subjected to corrosion studies.

## 2. Materials and Methods

### 2.1. Materials and Experiment details

Al 7005 alloy was used as a matrix material to fabricate the extruded parts. The S-glass fibres (possessing average fibre diameter: 5–10 µm) and fly ash (particle size of 25–30 µm) were used as reinforcement materials. Glass fibre with 5% wt. and fly ash of 6% wt. reinforcements to aluminium matrix resulted in better mechanical and wear resistance properties in the composites [65,66]. In addition, no casting defects and agglutination of reinforcements are observed with GF and FA kept fixed at 5% wt. and 6% wt., respectively. In general, minimal casting defects are less likely to undergo corrosion. Table 2 presents the chemical composition of reinforcement and matrix materials.

**Figure 1:**
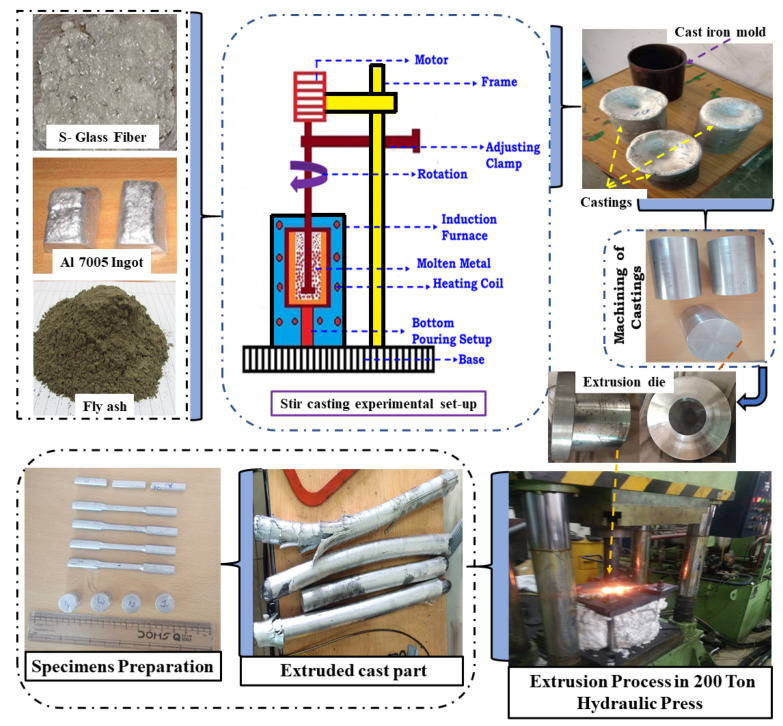
Flowchart illustrating the steps in fabricating the extrusion parts.

Four specimens (Al 7005, Al 7005 + 5%GF, Al 7005 + 6%FA, Al 7005 + 5%GF + 6%FA) were fabricated viz. stir casting technique. The Al 7005 ingots were melted in an electrical resistance crucible furnace with mechanical stirring attachments. The preheated (≈300 °C) reinforcement particles (S-glass fibre and fly ash) were added to the prepared melt (≈800 °C) and stirred continuously at 500 rpm, which ensures uniform dispersion in the Al 7005 matrix. The pre-mixed melt was allowed to pour into pre-heated die temperature (say, 200 °C) and allowed to solidify. The solidified four different specimens were extruded at 500 °C, subjected to different extrusion ratios kept fixed to 2.66:1 and 5.32:1, respectively. The rate of extrusion is maintained, equal to 0.5 mm/s. Figure 1 illustrates the steps involved in fabricating the extrusion products.

**Figure 2:**
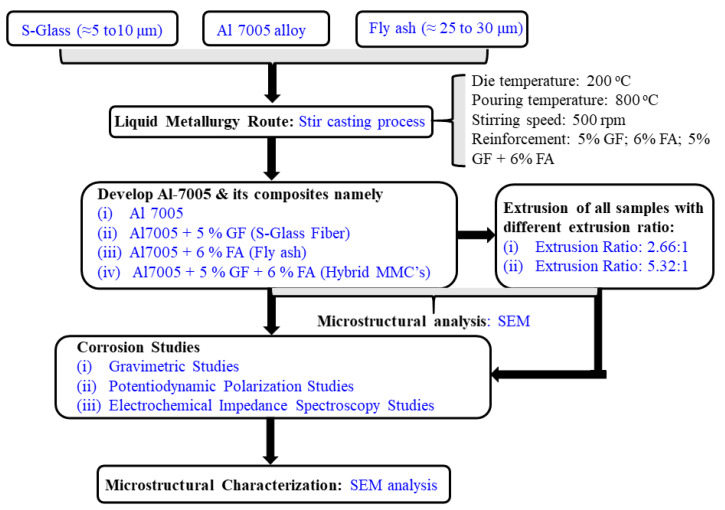
Flowchart illustrating the steps involved in the characterization of stir casting and extruded parts.

Four extruded samples (for each test sample, three replicate experiments were prepared) were polished (with series of sandpapers, followed by disc polishing with 1 μm diamond paste) and cleaned with water followed by acetone. Later, the specimens were air-dried. The polished specimens were etched with Keller’s solution (H_2_O + HNO_3_ + HF + HCl) to reveal the microstructure viz. scanning electron microscope. The framework of specimen preparation and characterization is presented in Figure 2.

### 2.2. Gravimetric Corrosion Tests

The polished test samples were washed in double distilled water, followed by air drying. The size of test samples with dimensions 15 mm × 15 mm × 5 mm was used for performing the weight loss method. The specimens were suspended in a beaker containing 250 mL of 1M HCl solution [70]. The 1M HCl solution is used for corrosion testing as it contains a higher percentage of chloride ions that serve the desired function as a passive film destabilizer [71]. To limit the evaporation of solution and contamination from surroundings, the corrosion vessel is sealed with paraffin. The samples are exposed to 1M HCl solution subjected to various time intervals of 24, 48, 72, 96, and 120 h (increment of 24 h) at room temperature. The choice of time intervals is selected after consulting the literature [72,73]. During weight loss measurements, the specimens’ weights are measured in an electronic digital weighing balance (possessing accuracy of 0.1 mg) before and after immersion in 1M HCl solution. After completing the required exposure time, the corroded specimens are taken out and cleaned by dipping the specimens in Clark’s solution (1L HCl + 20 g Sb_2_O_3_ + 50 g SnCl_2_) for 1 min [74], followed by scrubbing with a soft brush, washing with distilled water, and air-drying and weighing in the digital balance. For each sample, the gravimetric experiments were repeated thrice, and the average values were recorded to ensure reproducibility and performing precision analysis. The corrosion rates of samples are estimated using the mathematical expression given below [75],

(1) $$Corrossion\;rate=\frac{534\times w}{\rho \times A\times T}$$

Terms: Corrosion rate measured in mils per year (mpy), *w* is the weight loss in mg, *ρ* is the density in g/cm^3^, and *T* refers to exposure time in hours, while *A* is the surface area (lateral surface area + two circular face area) in inch^2^.

### 2.3. Electrochemical Measurements

The anti-corrosion performance of all test samples in 1 M HCl solutions was investigated viz. electrochemical tests (polarization potentiodynamic curves and electrochemical impedance spectroscopy EIS). The experimental set-up used for polarization measurements consisting of potentiostat/galvanostat (Model: CL-95, Elico Pvt. Ltd., Bengaluru, India), provided with a sweep generator and graphic (X-Y) plotter. The polished test samples with a surface area of 1 cm^2^, exposed to a 1M HCl electrolyte solution tested at room temperature. The experiments are conducted viz. three electrodes electrolytic cell, consisting of a counter electrode, i.e., platinum foil, reference electrode, i.e., Ag/AgCl (potential of +0.197 V at 25 °C), and test samples as the working electrode, respectively. The specimens are immersed in test solutions for at least a 30-min duration, ensuring that steady-state potential is achieved. The curves corresponding to potentiodynamic current are recorded for polarizing test specimens ± 250 mV, both cathodically and anodically. The tests are recorded at a sweep rate of 0.167 mV/s. The potentiostat variable (*I_corr_*: corrosion current in A/cm^2^, and *E_corr_*: corrosion potential) values are recorded to correspond to slopes obtained from polarization curves obtained from potentiostatic measurements. The expression to calculate the corrosion rate viz. potentiostatic measurements is presented in Equation (2) [76],

(2) $$Corrossion\;rate=\frac{1.287\times 10^{5}\times EW\times I_{Corr}}{D\times A}$$

Terms: *EW* (=atomic weight of sample/valence electron = 26.9815/3 g) refers to equivalent metal weight (g), *D* is the density (g/cm^3^) and *A* refers to exposed surface area (cm^2^).

The tests are conducted with an operating range between 1 to 10 mA after maintaining the fixed voltage of −300 mV and scan rate of 0.333 mV/s. EIS measurements are performed with a frequency range of 10 kHz^−1^ MHz using Solartron 1255 frequency response analyser (FRA).

## 3. Results and Discussion

This section discusses the comparison of the microstructure of as-cast and extruded parts with different extrusion ratios. Furthermore, the comparison of parts (as-cast and extruded with different ER) on corrosion rates are examined with gravimetric and electrochemical tests.

### 3.1. Microstructure Characterization of As-Cast and Extruded Samples

**Figure 3:**
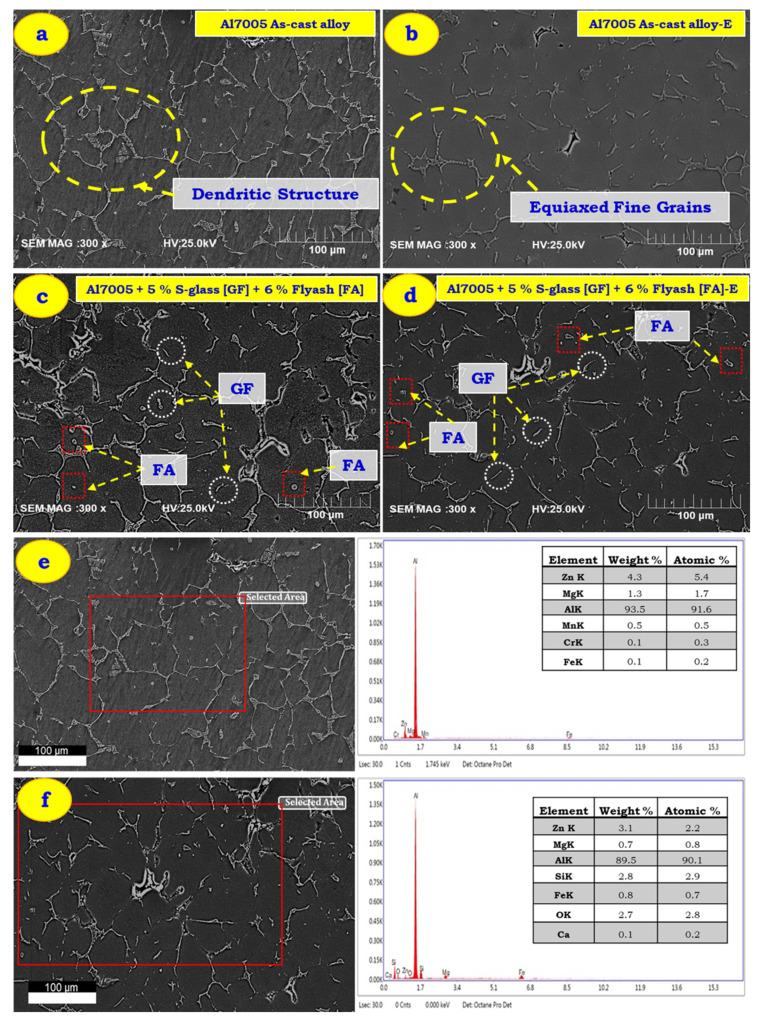
SEM of as-cast and extruded parts at ER: 5.32:1. (**a**,**b**) Al 7005 before and after extruded conditions, (**b**) Al 7005 + 5%GF, (**c**,**d**) Al 7005 + 6% FA + 5% GF before and after extruded conditions, (**e**) EDX of as-cast Al 7005, (**f**) EDX of Al 7005 + 6% FA + 5% GF after extrusion.

Figure 3 shows the morphologies of all four test samples (as-cast Al 7005, extruded Al 7005, as-cast Al 7005 + 5%GF + 6%FA, extruded Al 7005 + 6%FA + 5%GF) fabricated viz. stir casting and extrusion process (with an ER: 5.32:1).

The SEM morphology of Al 7005 alloy in as-cast and extruded conditions is presented in Figure 3a,b. Figure 3a shows clearly the presence of eutectic comprising of platelets with equiaxed shape grains in the matrix, revealing an Al-rich phase along with Zn and Mg as the primary alloying element. Figure 3b depicts the micrograph of Al 7005 subjected to the extruded condition, which reveals a network of consistent parallel sub-crystalline regions stretched along the perpendicular direction of extrusion, resulting in lengthy and refined grains. It was clear that as-cast Al 7005 samples subjected to the extrusion process result in a homogeneous and refined equiaxed grain in the microstructure. Figure 3c,d show the SEM morphology of Al 7005 with reinforced 5%GF and 6%FA in as-cast (before extrusion) and extruded conditions. The reinforced particles (GF and FA) are distributed randomly in an Al 7005 matrix which hinders the dendritic formation and their growth resulted in many fine-sized particles (refer to Figure 3c). More refinement with multiple fractures of particles as a result of better bonding developed between the reinforcement and the matrix was observed with the extruded sample (refer to Figure 3d). This occurs because the S-glass fibres tend to break due to reduction in cross-section as a result of stress developed wherein the matrix is squeezed to micro-cracks during the extrusion process. The extrusion die angle’s (i.e., higher diameter at the entrance and a smaller diameter at the exit) tendency to break the S-glass fibre may be due to stress in S-glass fibre transferred beyond its strength limit (refer to Figure 3). Similar observations are seen in TiB fibre-reinforced composites [31,77]. Figure 3e,f showed the EDS analysis of as-cast Al 7005 alloy and Al 7005 + 5% GF + 6% FA hybrid MMC’s. Figure 3e shows the peaks of Zn, Mg and Mn as the main alloying elements in the Al matrix which confirm the composition of Al 7005 as-cast alloy. From Figure 3f, the peaks of Si, Fe, O, Zn and Mg were observed along with Al, which confirms the presence of glass fibre and fly ash.

### 3.2. Gravimetric Corrosion Studies

**Figure 4:**
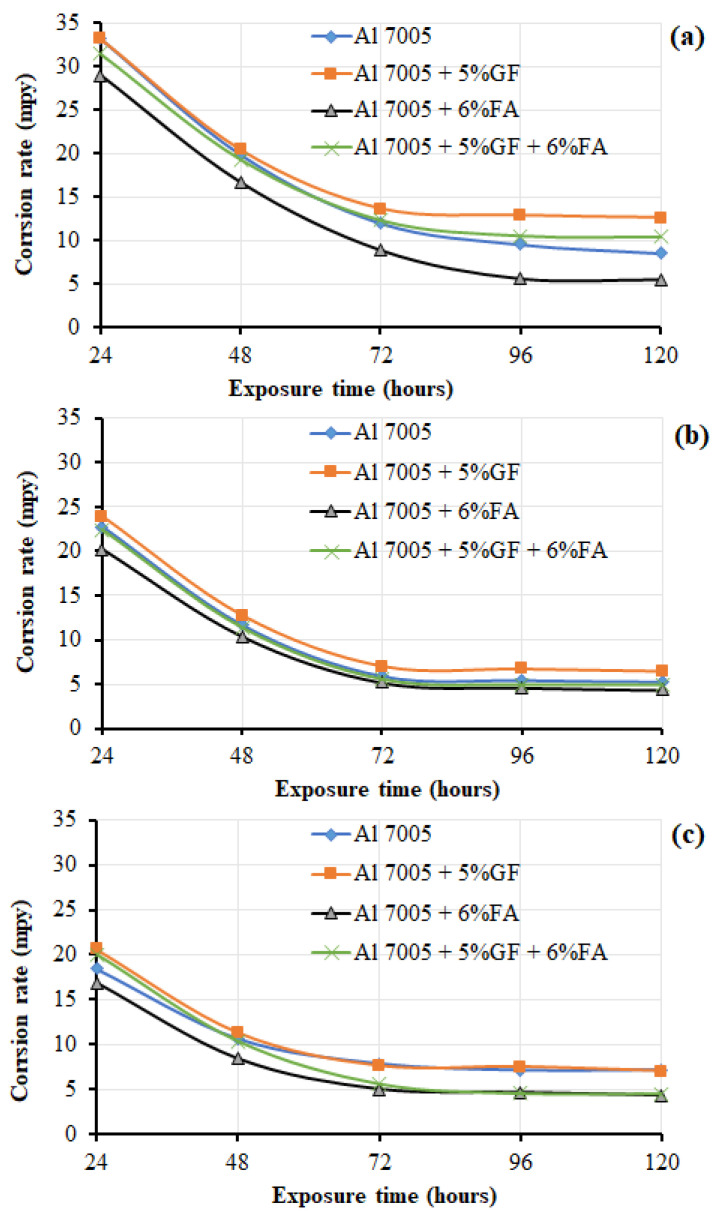
Effects of exposure time on the corrosion rate of Al 7005 and its composites: (**a**) as-cast, (**b**) 2.66:1 of ER and (**c**) 5.32:1 of ER.

Irrespective of chemical composition (Al 7005, Al 7005 + 5% GF, Al 7005 + 6% FA, Al 7005 + 5% GF + 6% FA) fabricated viz. stir casting and extrusion route, a decreasing trend in corrosion rate with increased duration of exposure in HCl media was shown. This might be due to the passivization on the corroded surface of the specimen. This is because the increased duration of exposure in HCl solutions tends to form the passive protective layer composed of hydrogen hydroxyl chloride film during the corrosion reaction. A similar trend was observed in corrosion studies of AA6082-T651 aluminium alloy subjected to NaCl solution [78]. Figure 4a–c clearly show the corrosion rate of as-cast Al 7005 and Al 7005 + 5% GF resulting in a higher corrosion rate due to the formation of pits, and crack formation on the corroded surface. GF in the aluminium matrix is seen to produce more cracks and discontinuities resulting in pits on the surface, which acts as a stress concentrator and potential site to promote corrosion resulting in a higher corrosion rate [71]. The corrosion rate is also influenced by the processing route, as stir cast parts contain a dendritic structure with enlarged grain boundaries associated with defects. These defects or discontinuities are reduced when subjected to hot extrusion wherein bonding between voids and discontinuities takes place subjected to higher pressure and temperature. This could result in an equiaxed structure with a lesser corrosion rate at extruded product compared to as-cast Al 7005 (refer to Figure 4a,b). Note that FA particles remain inert, which serve as a physical barrier (or do not react) with corrosive media during the corrosion test. Hence, the corrosion rates for Al 7005 + 6% FA composite are lower than that of all other materials (refer to Figure 4a–c). This might be due to fly ash particles protecting the matrix from pit formation and growth in the Al matrix [79].

Figure 4b,c clearly show that extruded parts offer greater resistance to corrosion for all specimens compared to as-cast or stir casting conditions. The corrosion rate variation in all extruded samples is comparatively lesser than that of as-cast samples. This could be due to the fact that casting defects such as porosity, voids, segregation and discontinuities are reduced and refined grain structures subjected to the extrusion process result in better corrosion resistance.

### 3.3. Potentiodynamic Polarization Studies

**Figure 5:**
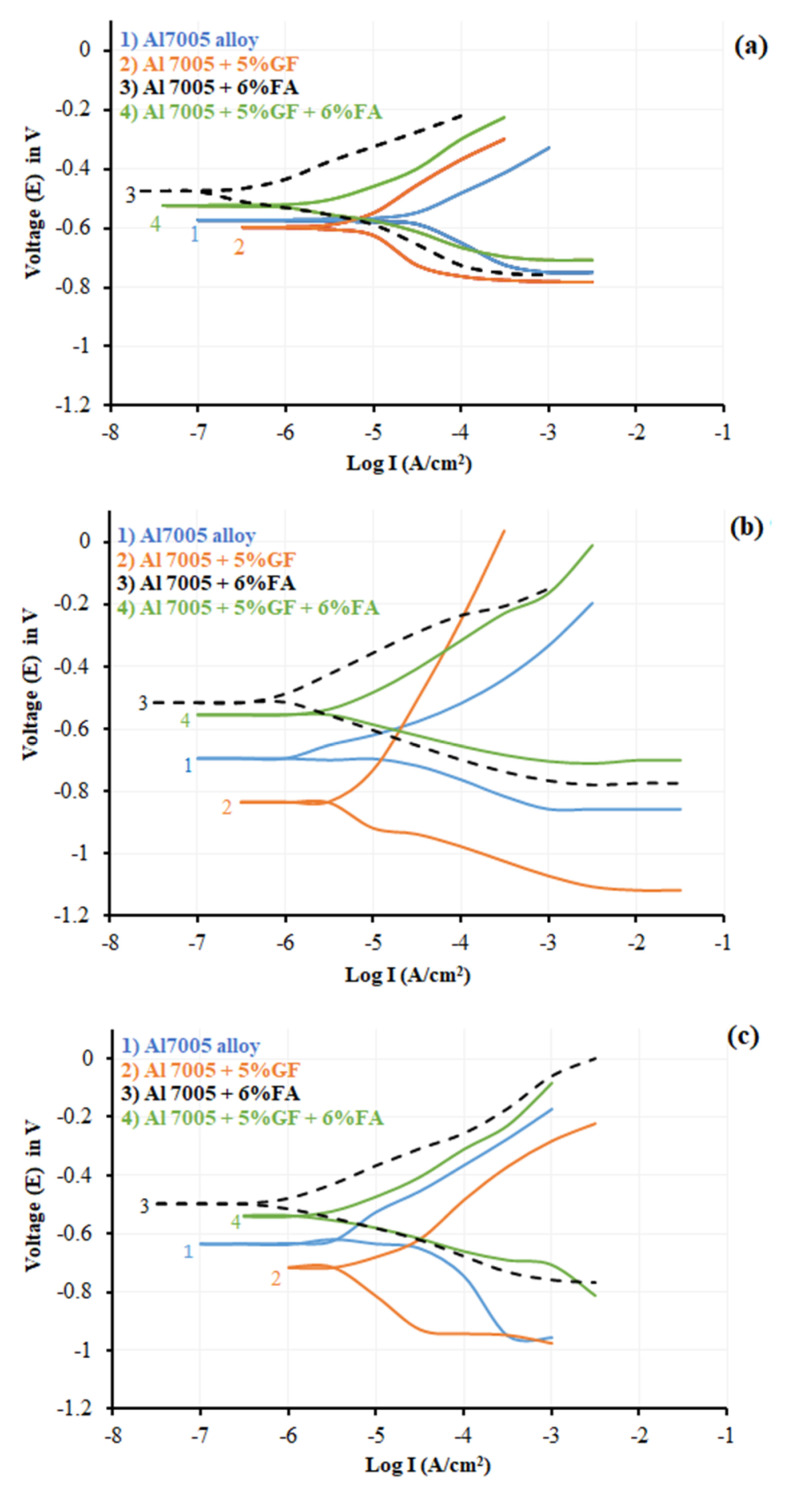
Polarization curves of Al 7075 and its composites: (**a**) as-cast, (**b**) ER: 2.66:1, and (**c**) ER: 5.32:1.

The polarization tests are carried out on both as-cast and extruded samples (with ER: 2.66:1 and 5.31:1) suspended in 1M concentration of HCl solutions at room temperature. The test results at different processing routes and extrusion ratio of all samples are presented in Figure 5a–c. The corrosion current density (*I_corr_*) and corrosion potential (*E_corr_*) were computed from the intersection of the tangent drawn for cathodic and anodic Tafel curves, presented in Table 3. In both as-cast and extruded conditions, the Al 7005 + 5% GF exhibited high destructive corrosion current which might be due to the combined effect of reduction and oxidation of electrochemical processes occurring at the interface of glass fibre and aluminium matrix. The low dielectric glass fibres are suspended for a prolonged time in HCl solution with 1M concentration during the electrochemical test, under fibre degradation or disruption of the continuity of glass network as a result of fibre leaching, hydrolysis, matrix plasticizing, and the fibre–matrix interface debonding phenomenon [80,81].

During testing the specimens, the hydrolysis phenomenon and electrochemical aggression occur simultaneously at the interface in the case of glass fibre-reinforced aluminium composites. An increase in voltage tends to damage the glass fibre and matrix interface severely. Hydrolysis acid creates pits, cracks, flakes and blisters that degrade the fibre–matrix interface [81]. Conversely, the corrosion current in fly ash-based composites is shown to be lower than that of other materials. The anodic curves of Al 7005 showed the continuity curves indicating the susceptibility of pitting corrosion. The fly ash particles improve the corrosion resistance to Al 7005 due to the following: FA particles remain inert or non-reactive, and fly ash absorbs the chlorine ions onto the oxide layer and produces a more stable layer on the aluminium alloy, and secondly, this alters the microstructure and they act as a protective barrier to corrosion damage and progression for pitting corrosion. FA particles in Al 7005 composites reduce the corrosion rate which makes the curves shift to a more active region. This occurs due to the formation of oxides around the fly ash particles and makes them neutral in the HCl environment and thereby the matrix material is cathodically protected from the acidic medium. Table 3 provides the details of computed values of the corrosion rates of both as-cast and extruded samples. For all the specimens irrespective of the presence of fly ash reinforcement, the corrosion rate decreases with decreases in the extrusion ratio.

### 3.4. Electrochemical Impedance Spectroscopy Studies

**Figure 6:**
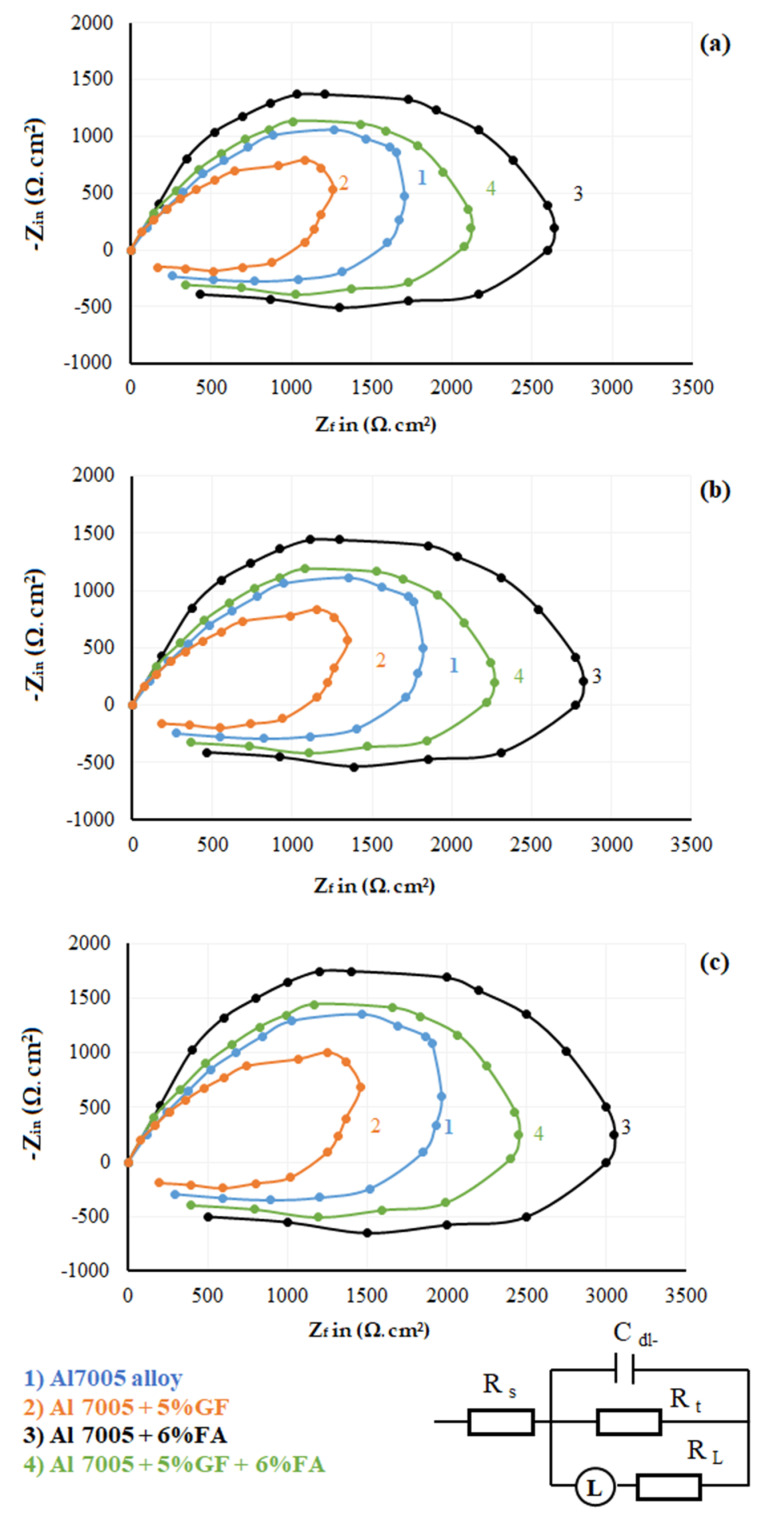
Nyquist plots of the Al 7075 and its composites: (**a**) as-cast, (**b**) ER: 2.66:1, and (**c**) ER: 5.32:1 Top right diagram shows Equivalent circuit diagram used for the experiment.

EIS measurements are carried out on all four samples subjected to as-cast and extruded conditions at open circuit potential shown at the right corner of Figure 6. In an open circuit, C_dl-_ corresponds to electrical double-layered capacitance at the Al 7005 and electrolyte interface, R_t_ is the charge transfer resistance, L is the inductance, R_L_ is the low-frequency loop resistance and R_s_ is the electrolyte resistance.

Figure 6a–c show that the Nyquist plots correspond to as-cast extrusion with ER: 2.66:1 and ER: 5.32:1 conditions of Al 7005, Al 7005 + 5% GF, Al 7005 + 6% FA, Al 7005 + 5% GF + 6% FA samples, respectively. The curves in Nyquist plots dictate the resistance of the electron transfer process corresponding to electrode surfaces. Note that, the larger the diameter of the arc, the greater the corrosion resistance. A larger diameter dictates that the loss/gain of electrons in anode and cathodes is more difficult which is often difficult to dissolve in bath solution (i.e., 1M HCl solution), resulting in a decrease in the corrosion rate of samples [82]. From Figure 6a, the corrosion rates are minimum with 6% FA addition to Al 7005, but it is maximum with 5% GF addition to Al 7005, and the rest of the specimen lies between them. Irrespective of the processing route (stir cast or extrusion), there is a continuous increase in the diameter of Nyquist circles with the addition of FA particles in the Al 7005 matrix material. This suggests that the presence of an inhibitor gradually changed the corrosion reactions on the electrode surface. All Figure 6a–c show imperfect semicircle structures which appear with one smaller than the other. The size of the imperfect semicircle diameter is influenced by the presence of a type of reinforcement and extrusion ratio on the corrosion of Al 7005 inhibition. Higher diameter curves are observed with 6% FA reinforced to Al 7005 alloy (i.e., Al 7005 + 6% FA). FA addition ensures insulation of metal and solution interface by creating the surface film. This film contributes to an increase in charge transfer resistance, which offers higher corrosion resistance. The imperfection shape of the semicircle may be attributed to the surface roughness of the specimen [83]. The roughness of the specimens increases with the addition of glass fibre to composites which causes adsorption of electrolyte solution molecules to the active sites of the composite surface and reduces the charge transfer resistance results in a higher corrosion rate. The higher ER of 5.32:1 made a wider imperfect semicircle than the stir casting condition, which reduces the corrosion rate in all the specimens.

**Figure 7:**
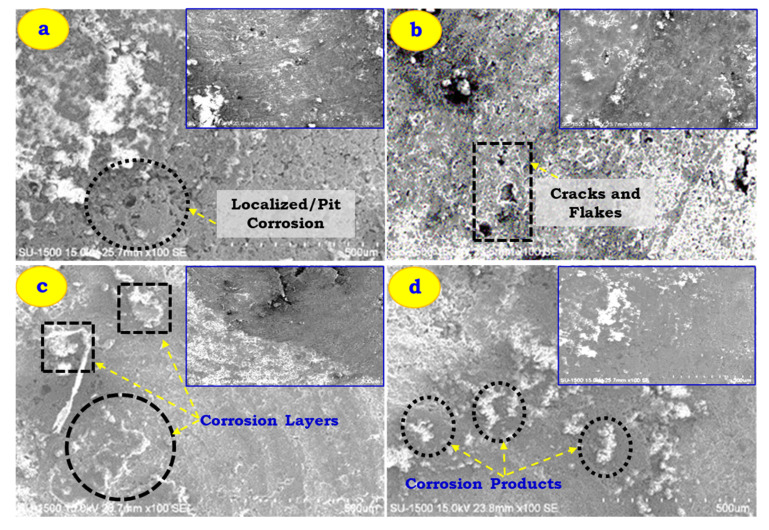
Corrosion morphology in as-cast and extruded (ER: 5.32:1) Al 7005 and its composites immersed in 120 h in 1 M HCl solution (right top image are not extruded corroded samples) (**a**) Al 7005, (**b**) Al 7005 + 5% GF, (**c**) Al 7005 + 6% FA and (**d**) Al 7005 + 6% FA + 5% GF.

Figure 7 shows the corrosion morphology of Al 7005 cast and composite specimens (as-cast and extruded) in HCl solution for 120 h. Irrespective of the processing route, in all the as-cast and extruded specimens, there exists a pit-type corrosion (i.e., localized corrosion) and white corrosion products are observed on the surface of the specimen (refer to Figure 7a). The corroded product shows aluminium hydroxide which degrades the surface of the specimen, which can be seen as an as-deposited surface. As the specimen is immersed in a solution for a prolonged duration, more corrosion products get deposited which causes more cracks and flakes to appear on the surface (refer to Figure 7b). More corrosion product appears on the as-cast Al 7005 and Al 7005 + 5% GF, which might be due to cracks and discontinuities that appear more on the surface than other samples (Al 7005 + 6% FA, Al 7005 + 6% FA + 5% GF). In a hot extrusion product, the cracks and discontinuities are fused, which inhibits the corrosive medium to penetrate and therefore offers resistance to corrosion. Therefore, extruded products offer a more protective layer on the surface and prevent corrosion. Glass fibre reinforced to Al 7005 results in the formation of pits around the particles, and the corresponding area is the potential site to initiate and propagate the corrosion. Therefore, more corrosion products are seen in Figure 7b. The hydrogen bubbles are liberated during corrosion, which breaks the protective layer in the forming mouth of a volcano. Fly ash reinforced to Al 7005 alloy tends to fill the voids, cracks, and discontinuities, and thereby corrosion products are less comparable to as-cast Al 7005, and Al 7005 + 5% GF composites (refer to Figure 7c). Figure 7d represents the corrosion morphology of Al 7005 + 5% GF + 6% FA, showing a similar trend with few more layers of corrosion than that obtained for Al 7005 + 6% FA. This occurs because of tiny micro-cracks produced around the surface layers of glass fibre. These cracks connect and cause the progressive removal of structure on the surface as shown in Figure 7. This is commonly called flaking. SEM of the flakes, which were formed from the corroded sample, as well as the flakes remaining in the sample, was taken (refer to Figure 7b). It can be concluded that the fly ash reinforced to Al 7005 resulted in fewer corrosion products than other samples.

## 4. Conclusions

The present work uses industrial wastes (fly ash and glass fibres) as potential reinforcement materials for fabricating composites, which offer beneficial properties useful for structural applications. Thereby, the present work conducts experimental studies to examine the corrosion behaviour of Al 7005 and its composites fabricated viz. stir casting and extrusion process route. Corrosion studies are carried out on all specimens under 1 M HCl environment, using gravimetric, electrochemical and impedance studies. The following conclusions are drawn from the present experimental investigation,

1. The presence of voids or porosities were observed in Al 7005 alloy stir cast conditions, which are reduced subjected to extrusion pressure. The glass fibre breaks and refines the grain structure of as-cast composite (Al 7005 + 5% GF + 6% FA) parts subjected to extrusion.
2. The gravimetric corrosion behaviour of Al 7005 and its composites in an HCl environment showed decreased corrosion rate with increased testing duration, due to the passive layer deposited on the surface of the specimen. The corrosion rate of Al 7005 composites showed mixed behaviour for fly ash with a lower corrosion rate, but higher in the case of glass fibre.
3. Polarization potentiodynamic studies showed that Al 7005 resulted in the highest corrosion rate, followed by Al 7005 + 5% GF, Al 7005 + 5% GF + 6% FA, and Al 7005 + 6% FA. High destructive corrosion current was observed with GF reinforcement, which might be due to the synergetic effect of reduction and oxidation of electrochemical process occurs at the interface of glass fibre and aluminium matrix. Furthermore, GF undergoes fibre degradation or disrupts the continuity of the glass network as a result of fibre leaching, hydrolysis and the fibre matrix interface debonding phenomenon.
4. FA particles reinforced to the aluminium matrix showed improved corrosion resistance property, which might be due to the gaps or discontinuities in the form of pits or cracks filled with FA. These FA particles act as a potential site to resist corrosion by creating a surface film.
5. The corrosion rate increasing with GF might be due to the formation of pits or discontinuities around the fibre particle. The area around the GF particle serves as a potential pit-initiating site.
6. The impedance studies show the same nature of the behaviour of gravimetric corrosion, but a lesser significant change in corrosion behaviour. Higher diameter curves are observed with 6% FA reinforced to Al 7005 alloy (i.e., Al 7005 + 6% FA) which ensures higher corrosion resistance. FA addition ensures insulation of metal and solution interface by creating the surface film. This film contributes towards an increase in charge transfer resistance, which offers higher corrosion resistance.
7. The corrosion morphology study reveals that the corrosion layer, pits, cracks, and flakes are major contributors to the removal of material from the host material during corrosion testing. Al 7005 + 6% FA resulted in lesser corrosion products than that obtained for other samples.

## Figures and Tables

**Table 1:** Summary of literature review of extrusion of aluminium alloys and their characterizations.

Matrix	Reinforcement Particles and Amount	Size of Reinforcements	Remark	Reference
Pure Al	SiC and 0–1.5% vol.	15 nm and ER: 20.25:1	↑ 121% H and ↑ 11% TS	[43]
Pure Al	Al_2_O_3_ and 5–15% vol.	45 µm and ER: 20.25:1	↑ 59.78% H and ↑ 24.67% TS	[44]
Pure Al	MWCNT and 2% wt.	140 ± 30 nm outer ⌀, 4–8 nm inner ⌀, and ER: 4:1	↑ 3 times higher in H and ↑ 21% higher in TS	[45]
Al2024	Al_18_B_4_O_33_ and 25% vol.	⌀: 0.5–1 µm, length 10–20 µm, ER: 9:1, 16:1, 25:1	↑ 2 times higher in TS, refined grained structure	[24]
Al–Zn–Mg–Cu composite	TiB_2_ and 6% wt.	*<*100 nm	↑ 482 MPa to 687 MPa in TS and ↑ ductility from 2% to 14.8%.	[23]
Pure Al	SiC and 0.3–1.5% vol.	15 nm and ER: 20.25:1	↑ H from 37 to 86 Hv, CS from 323 to 373 MPa, TS from 133 to 184 MPa	[46]
Pure Al	MWCNT, GNPs and C60 and 0.25% wt.	8–18 nm of MWCNT, 1.5 µm of GNPs, and ER: 16:1	↑ H by 17%, 22% and 26% with added MWCNT, GNPs and C60, ↑ TS by 27%, 33% and 48% with added MWCNT, GNPs and C60	[47]
Al 6061	BN, and 6–9% wt.	ER: 3.06:1	↑ H by 17%, ↑ TS by 18.9%	[48]
Pure Al	G and 1% wt.	0.5–20 µm and ER: 9:1	↑ H from 37 to 70 VHN, ↓ GS from 30 to 24 µm, ↑ TS by 46%	[25]
Pure Al	SiC and 5–30% wt., Al_2_O_3_ and 5–25% wt.	SiC: 300 µm, Al_2_O_3_: 90 µm and ER: 16:1	↑ H and WR was improved	[49]

SiC: silicon carbide; H: hardness; TS: tensile strength; Al_2_O_3_: aluminium oxide; MWCNT: multi-wall carbon nanotube; TiB_2_: titanium diboride; Al_18_B_4_O_33_: aluminium borate; CS: compression strength; G: graphene; GNPs: graphene nanoplatelets; C60: carbon; BN: boron nitride; YS: yield strength; WR: wear resistance.

**Table 2:** Chemical composition of matrix and reinforcement materials.

S-Glass [67]		Fly Ash [68]		Al 7005 [69]	
Elements	Wt.%	Elements	Wt.%	Elements	Wt.%
Al_2_O_3_	26	Al_2_O_3_	29.6	Zn	4.44
MgO	10	CaO	0.10	Mg	1.38
SiO_2_	64	Fe_2_O_3_	0.72	Mn	0.54
-	-	K_2_O	3.53	Cr	0.10
-	-	MgO	0.34	Fe	0.11
-	-	SiO_2_	64.6	Si	0.03
-	-	-	-	Cu	0.01
-	-	-	-	Al	Bal.

**Table 3:** Icor and corrosion rate of extruded and non-extruded Al composites.

Extrusion Condition	Test Samples			
	Al 7005	Al 7005 + 5% GF	Al 7005 + 6% FA	Al 7005 + 6% FA + 5% GF
	*I_corr_* (mA/cm^2^)			
**Before Extrusion**	31.623	7.079	3.162	5.129
**2.66:1 Extrusion**	7.943	6.310	1.230	2.512
**5.32:1 Extrusion**	7.586	5.623	0.794	1.778
	Corrosion rate, mpy			
**Before Extrusion**	13.56 ± 0.30	3.04 ± 0.12	1.36 ± 0.22	2.20 ± 0.11
**2.66:1 Extrusion**	3.41 ± 0.25	2.70 ± 0.15	0.53 ± 0.12	1.08 ± 0.14
**5.32:1 Extrusion**	3.25 ± 0.23	2.41 ± 0.12	0.34 ± 0.08	0.76 ± 0.14

## Data Availability

Data Sharing is not applicable.
